# A two-stage hybrid biomarker selection method based on ensemble filter and binary differential evolution incorporating binary African vultures optimization

**DOI:** 10.1186/s12859-023-05247-7

**Published:** 2023-04-04

**Authors:** Wei Li, Yuhuan Chi, Kun Yu, Weidong Xie

**Affiliations:** 1 Key Laboratory of Intelligent Computing in Medical Image (MIIC), Northeastern University, Ministry of Education, Shenyang, China; 2 School of Computer Science and Engineering, Northeastern University, Shenyang, China; 3 School of Biomedical and Information Engineering, Northeastern University, Shenyang, China

**Keywords:** Feature selection, Ensemble filter method, Binary differential evolution, African vultures optimization, Microarray data

## Abstract

**Background:**

In the field of genomics and personalized medicine, it is a key issue to find biomarkers directly related to the diagnosis of specific diseases from high-throughput gene microarray data. Feature selection technology can discover biomarkers with disease classification information.

**Results:**

We use support vector machines as classifiers and use the five-fold cross-validation average classification accuracy, recall, precision and F1 score as evaluation metrics to evaluate the identified biomarkers. Experimental results show classification accuracy above 0.93, recall above 0.92, precision above 0.91, and F1 score above 0.94 on eight microarray datasets.

**Method:**

This paper proposes a two-stage hybrid biomarker selection method based on ensemble filter and binary differential evolution incorporating binary African vultures optimization (EF-BDBA), which can effectively reduce the dimension of microarray data and obtain optimal biomarkers. In the first stage, we propose an ensemble filter feature selection method. The method combines an improved fast correlation-based filter algorithm with Fisher score. obviously redundant and irrelevant features can be filtered out to initially reduce the dimensionality of the microarray data. In the second stage, the optimal feature subset is selected using an improved binary differential evolution incorporating an improved binary African vultures optimization algorithm. The African vultures optimization algorithm has excellent global optimization ability. It has not been systematically applied to feature selection problems, especially for gene microarray data. We combine it with a differential evolution algorithm to improve population diversity.

**Conclusion:**

Compared with traditional feature selection methods and advanced hybrid methods, the proposed method achieves higher classification accuracy and identifies excellent biomarkers while retaining fewer features. The experimental results demonstrate the effectiveness and advancement of our proposed algorithmic model.

## Background

In bioinformatics, DNA microarray technology can obtain a large number of gene expressions at once time, but only a few of these genes are directly relevant to the diagnosis and prediction of specific diseases, which we refer to as biomarkers. It is important to investigate how biomarkers can be discovered and modeled for classification. However, gene microarray data has the characteristics of high dimensionality, few samples and high redundancy that traditional machine learning models cannot be directly used for microarray data mining, so researchers usually use feature selection technology to discover biomarkers in gene microarray data [1]. The purpose of feature selection is to select a low dimensional feature subset that can distinguish specific diseases [2]. According to different evaluation criteria, feature selection methods can usually be divided into three categories, namely filter method, wrapped method, and embedded method.The filter method uses general statistical attributes to individually evaluate each feature. The wrapped method usually uses evolutionary or biological heuristic algorithms to guide the search process, and the accuracy calculated by a specific classifier is used to evaluate the selected feature subset. The embedded method integrates the feature selection mechanism into the training process of the learning model and automatically selects features during model training [3]. Although the filter method has a low computational cost and fast running speed, it ignores the feature interaction and the performance of the selected feature on the classification algorithm. The wrapped method can achieve the highest classification accuracy, but the amount of calculation is large. The classification accuracy of the embedded method is better than the filter method but not as good as the wrapped method, and the time complexity is better than the wrapped method but not as good as the filter method, and the learning model used is highly dependent on the parameters [4].

According to the current study, a single feature selection method cannot effectively identify significant features. However, the application of hybrid feature selection methods to extract the most informative genes has achieved good results [5]. The hybrid method can be a combination of two different methods such as the filter method and the wrapped method, two methods of the same criterion, or two feature selection approaches [6]. Compared with the single feature selection method, the hybrid method provides better accuracy and computational complexity and it is not easy to overfit [7]. Recently, many excellent hybrid feature selection algorithms have been used to select biomarkers in microarray data. These methods combine the advantages of different feature selection techniques to efficiently discover feature subsets.

Almutiri et al. proposed a feature selection method based on chi-square statistic and support vector machine (SVM) with recursive feature elimination (SVMRFE). In the first stage, chi-square was used to calculate the correlation between genes and category labels, and the top 10% genes in the correlation degree were used as candidate subsets, and SVMRFE was used to further select 10 genes with rich classification information. The experimental results proved that the classification results are improved compared to the methods in previous studies [8]. Al-Wajih et al. proposed a memetic method called HBGWOHHO, which mixes the binary gray wolf optimizer and harris hawks optimization, and uses the sigmoid function to convert the continuous search space into a binary space. And a wrapped-based k-nearest is used to evaluate the quality of the selected features [9]. Anter et al. designed a hybrid crow search optimization algorithm that fuses chaos theory and fuzzy c-means algorithm, called CFCSA, for the feature selection problem of medical diagnosis [10]. Mahapatra et al. proposed a two-staged hybrid arrangement of model, called mRMR-SSA. In the first stage, a preliminary dimensionality reduction of the dataset is performed using the filter method mRMR, and the resulting feature subset is input into the wrapped method SSA in the next stage. Measure model performance using classifiers such as XGBoost, AdaBoost, Random Forest, and Logistic Regression. [11]. Alomari et al. proposed a two-stage gene selection method based on the min-Redundancy and max-Relevance and Bat algorithm [12]. Pino et al. proposed an algorithm, called Gbc, which uses a combination of genetic algorithm and bee colony algorithm to search for the optimal solution [13]. Yu et al. proposed a hybrid biomarker discovery algorithm, called ILRC. First, the features were clustered to remove redundant features in subclusters. Then, all remaining features are iteratively evaluated using ILR. Reorder the features according to the accumulated weights to get the final result [14]. El-Hasnony proposed a hybrid feature selection algorithm based on butterfly optimization algorithm and particle swarm optimization algorithm. The proposed method is evaluated using the COVID-19 dataset. Compared with previous methods, the results show that this method has advantages in improving performance accuracy and minimizing the number of selected features [15]. Wang et al. proposed a hybrid feature selection method named MMPSO. By combining feature ranking methods and heuristic search methods, the best subsets that can be used for higher classification accuracy are obtained. The superiority of this approach was demonstrated by analyzing ten datasets obtained from the UCI Machine Learning Repository with a biological dataset containing gene expression information about liver hepatocellular carcinoma samples [16]. Wu et al. proposed a hybrid improved binary quantum particle swarm optimization algorithm, called HI-BQPSO, for feature selection problems. Combining the filter method with the improved quantum-behavior particle swarm optimization algorithm greatly reduces the dimensionality of the data [17]. Dong et al. proposed a feature selection algorithm model based on granularity information and tested how the granularity level affects the classification accuracy and the size of the selected feature subset through experiments [18]. Gao et al. proposed a hybrid method based on information gain and SVM to select biomarkers, and they obtained better classification accuracy [19]. Wu et al. proposed two-stage sequential minimum optimization to reduce the computational cost of large-scale training data, and proposed two-stage differential learning particle swarm optimization to ensure the accuracy of under-sampled data [20]. Vanitha et al. used SVM and gene selection methods based on mutual information to classify gene expression data, which effectively improved the classification accuracy [21]. Sadeghian et al. proposed a three-stage feature selection method, named EIT-bBOA. In the first stage, The method uses the minimal redundancy-maximum new classification information method to remove 80 percent of irrelevant and redundant features. In the second stage, the best feature subset is selected using the designed information gain binary butterfly optimization algorithm. Finally, a similarity based ranking method is used to obtain the final feature subset [22].

However, we discover that the following problems usually exist in the current research on hybrid feature selection algorithms:

- Researchers usually use a single filter method to perform initial dimensionality reduction on microarray data. This method lacks stability and ignores the interaction between features, potentially filtering out excellent features.
- Although these studies can achieve high classification accuracy, they do not pay attention to controlling the number of retained features, which may contain redundant features.
- Microarray data has continuity, but the situation in which the spatial search process is in a discrete state is generally ignored.

In the previous work, we proposed a two-stage hybrid feature selection method, named MMBDE [23]. In the first stage, mRMR was used to perform initial dimensionality reduction on the original data. In the second stage, an improved binary differential evolution algorithm was used to select the optimal feature subset, and identify excellent biomarkers. We validate the effectiveness of MMBDE on Leukemia, Lymphoma, Colon and Prostate datasets, and the experimental results show that the method can identify biomarkers closely related to disease.

In this paper, we further improve the MMBDE algorithm and propose a new two-stage feature selection method. In the first stage, an ensemble filter method is used to perform preliminary dimensionality reduction on the microarray data to obtain a stable and excellent candidate feature pool. In the second stage, the binary differential evolution algorithm was further improved, and the African vulture optimization was added to it to improve the performance of the algorithm. Experimental results demonstrate that the propose method in this paper outperforms MMBDE on most datasets. The main contributions of our paper are the following:

- An ensemble filter method that combines an improved fast correlation-based filter method with fisher score is proposed. It can perform preliminary dimensionality reduction on microarray data and provide an excellent candidate feature pool for downstream wrapped method.
- A hybrid wrapped method, BDBA, is proposed, which is binary differential evolution incorporating binary African vultures optimization. And a segmental adaptive fitness calculation method is designed. High classification accuracy can be obtained while retaining a small number of features.
- The evolution operator of binary differential evolution algorithm is improved. The African vultures optimization algorithm (AVOA) is binarily quantized, and its best vulture selection strategy is improved. so that they can deal with feature selection problems in discrete spaces.

The rest of this paper is organized as follows. The “Results” part provides experimental results with analysis and comparison. The “Discussion” part describes fully the proposed two-stage feature selection algorithm. Finally, the “Conclusion” parts concludes this paper.

## Results

### Evaluation and analysis of ensemble filter method

**Fig. 1:**
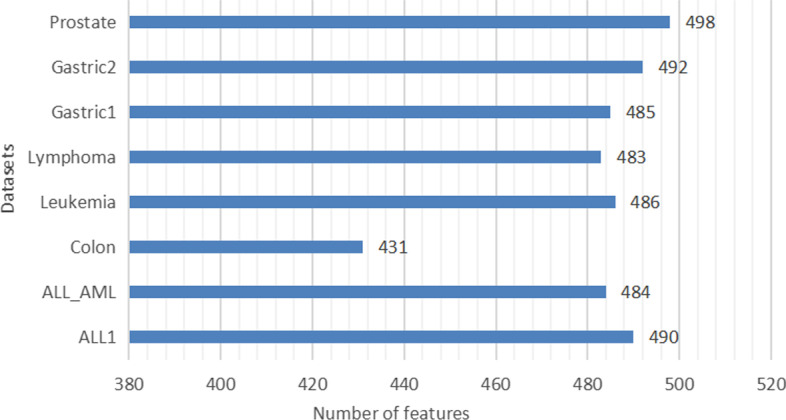
The number of features retained by ensemble filter method on each of the eight public datasets. The fisher score and the improved mRMR retain the same number of features

**Fig. 2:**
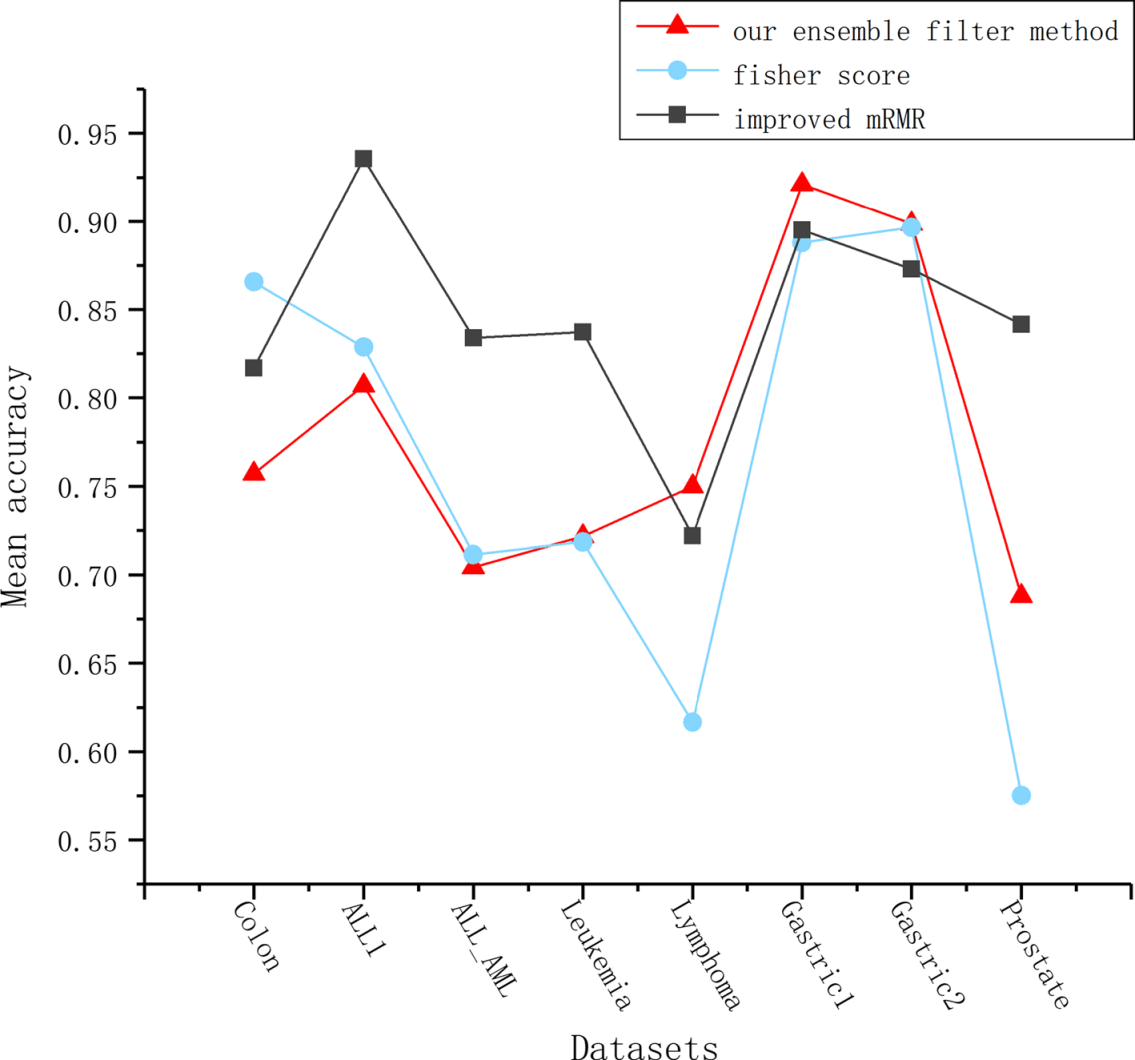
Compare the quality of candidate feature pools obtained by our ensemble filter method, fisher score and improved mRMR. Use Lasso regression, random forest, Linear regression, and chi-square test to perform feature selection in the two feature pools respectively, and then use the SVM classification model to perform five-fold cross-validation on the feature data selected by each model. Finally, the average classification accuracy obtained by the four models is used as the evaluation standard

To demonstrate the effectiveness of the proposed ensemble filter method, we make the single fisher score method and the mRMR method used in MMBDE as comparative experiments. Specifically, three feature selection methods are used on eight datasets, then each method yields eight feature pools. To ensure fairness, we make each method obtain the same dimensionality of the feature pool on the same dataset. Since the dimensionality of the feature pool obtained by the ensemble filter method is unpredictable, while the remaining two methods can intercept a subset of features with specified dimensionality based on feature ranking. Therefore, we use the feature pool dimension obtained by the ensemble filter method as a criterion. The specific dimensional information is shown in Fig. 1. Then, for each dataset, four traditional methods are used to measure the quality of the feature pools obtained by the three filter methods. The four traditional methods include Lasso regression, random forest, Linear regression, and chi-square test. This is done by retaining the top ten feature subsets in each feature pool using each of the four traditional methods. Then, a SVM is used on the data corresponding to each feature subset to obtain a five-fold cross-validation classification accuracy. Finally, the performance of the three filtering methods is measured by the average classification accuracy. The specific experimental results are shown in Fig. 2.

Among the eight datasets, our ensemble filter method outperformed fisher score on five datasets. On the Lymphoma, Gastric1 and Prostate datasets, the average classification accuracy corresponding to the feature pool obtained by ensemble filter method was significantly higher than fisher score, while on the Leukemia and Gastric2 datasets, the ensemble filter method outperforms fisher score by a small margin. Only on the Colon and ALL1 datasets, the feature pool filtered by fisher score is better than the ensemble filter method, while on the ALL_AML dataset, the fisher score advantage is not obvious. In general, the ensemble filter method outperforms fisher score alone on more than half of the datasets, and achieves an overall average classification accuracy $2\%$ higher than the fisher score using the four traditional methods on eight datasets. We have carefully improved the mRMR algorithm in MMBDE, so that it can obtain a higher quality feature pool. However, one obvious drawback of this algorithm is the very high time complexity, caused by the need to compute the correlation for each pair of features. From Fig. 2, we can see that the ensemble filter method does not perform as well as the improved mRMR on most of the datasets, but the execution efficiency of the ensemble filter method is far higher than the mRMR. Under the same conditions, the execution time of the improved mRMR method is at least 80 times longer than the ensemble filter method when the number of features in the dataset exceeds ten thousand. Our ultimate goal is to identify meaningful biomarkers. The wrapped method, as the major part of EF-BDBA, can effectively improve the overall classification accuracy of the algorithm. It is unnecessary to consume too much time on the filter method. Therefore, we use the ensemble filter method in EF-BDBA instead of the improved mRMR method.

### Evaluation and analysis of BDBA method

In this subsection, we analyze the adaptive crossover factor parameter $\alpha$ of the BDBA method, and compare our method with the traditional binary difference evolution method.

#### Comparison of adaptive cross factor parameter at different stages

In BDBA, the adaptive cross factor $\alpha$ is the only parameter with adjustable space in the evolution operator. Through comparative experimental analysis of $\alpha$ with different stage values, using classification accuracy and number of retained features as evaluation criteria, we found that $\alpha = 0.9$ performed the best overall. The specific experimental results are shown in Table 1.

**Table 1:** Influence of adaptive cross factor parameter on BDBA

Parameter	Datasets	Features	Accuracy
$\alpha =1$	Colon	5	0.9333
	ALL1	4	0.9923
	ALL_AML	3	0.9581
	Leukemia	4	0.9714
	Lymphoma	3	0.9778
	Gastric1	3	0.9510
	Gastric2	3	0.9673
	Prostate	4	0.9210
$\alpha =0.9$	Colon	3	**0.9346**
	ALL1	4	**1.0000**
	ALL_AML	4	0.9857
	Leukemia	3	**0.9714**
	Lymphoma	3	0.9778
	Gastric1	4	**0.9717**
	Gastric2	4	**0.9840**
	Prostate	4	**0.9405**
$\alpha =0.5$	Colon	7	0.9179
	ALL1	5	1.0000
	ALL_AML	6	0.9714
	Leukemia	5	**0.9714**
	Lymphoma	4	**1.0000**
	Gastric1	8	0.9648
	Gastric2	5	0.9840
	Prostate	8	0.9214
$\alpha =0.1$	Colon	5	0.9192
	ALL1	9	1.0000
	ALL_AML	6	**0.9867**
	Leukemia	10	0.9581
	Lymphoma	3	0.9778
	Gastric1	8	0.9645
	Gastric2	7	0.9840
	Prostate	4	0.9210

In Table 1, the bold indicates the highest classification accuracy obtained by BDBA when α is taken at different values on the same dataset. When $\alpha =0.9$, BDBA has the highest classification accuracy on the six datasets, and the number of retained features is relatively small, and the overall performance is the best. However, it is worth noting that as $\alpha$ decreases, the number of features retained by the algorithm does not increase on all datasets. It is only due to the difficulty of population mutation that the proposed mutation operator cannot effectively control the number of features retained. It increases the probability that the optimal individual dimension on some datasets becomes larger. In addition, it is not that the more features are retained, the higher the classification accuracy will be, because it is very likely that the increased features are redundant features without classification information. Taking extreme contrasts $\alpha = 1$ and $\alpha = 0.1$ as examples, as the number of iterations increases, CR decreases in the intervals (1, 0.23) and (0.1, 0.023), respectively. When $\alpha =0.1$, it is difficult for individuals to mutate. Since more individuals are retained when the population is randomly initialized, the number of retained features is larger compared to $\alpha =1$, as shown in the results on the ALL1 and Leukemia. However, because our proposed segmental adaptation fitness calculation method also controls the number of features, datasets such as Lymphoma and Prostate do not seem to be affected by $\alpha$. The reason why the result is the best when $\alpha =0.9$ is that individual variation is not difficult, and compared with $\alpha =1$, the risk of premature maturity of the algorithm is reduced, so the classification accuracy is higher and the number of retained features is less.

#### Comparison with traditional binary differential evolution method

Since the differential evolutionary algorithm is not suitable for dealing with discrete-space optimization-seeking problems, the traditional binary differential evolution method uses a sigmoid function to discretize the solution set. To ensure the fairness of the comparison experiments, the values of the common parameters (maximum number of iterations *G* and population size *NP*) of our BDBA and traditional binary differential evolution method were kept consistent. In addition, the scaling factor F and crossover factor CR of the traditional binary differential evolution method take the common value of 0.5.

As can be seen in Table 2, the traditional binary differential evolution method retains an excessive number of features. It performs less well than BDBA on the Colon, ALL_AML, Gastric1 and Prostate datasets.The classification accuracy of traditional binary differential evolution method is the same as BDBA on the ALL1 dataset, but the number of selected features is much larger than BDBA. The traditional binary differential evolution method had slightly better classification accuracy than BDBA on the Leukemia, Lymphoma and Gastric2 datasets, but the number of retained features was at least 15 times higher than that of BDBA.

**Table 2:** Classification accuracy of BDBA and traditional binary differential evolution (traditional BDE) on microarray data

Datasets	Traditional BDE		BDBA	
	Features	Accuracy	Features	Accuracy
Colon	49	0.9333	3	0.9346
ALL1	59	1.0000	4	1.0000
ALL_AML	59	0.9867	4	0.9875
Leukemia	68	1.0000	3	0.9714
Lymphoma	45	1.0000	3	0.9778
Gastric1	57	0.9645	4	0.9717
Gastric2	61	1.0000	4	0.9840
Prostate	61	0.9314	4	0.9405

### Evaluation and analysis of EF-BDBA method

In this subsection, we evaluate and analyze EF-BDBA. Firstly, we test its performance using the classical evaluation criteria, secondly, we compare it with four traditional feature selection methods. Thirdly compare EF-BDBA with advanced feature selection methods. Finally, we do statistical analysis on the biomarkers identified by EF-BDBA.

#### Performance evalution of EF-BDBA

Since BDBA performs best overall when $\alpha =0.9$, we evaluate the performance of EF-BDBA at $\alpha =0.9$. We test the performance of EF-BDBA using SVM-based five-fold cross-validation classification accuracy, recall, precision and F1 score as evolution metrics. The specific results are shown in Table 3.

**Table 3:** Performance evaluation of EF-BDBA($\alpha =0.9$)

Datasets	Accuracy	Recall	Precision	F1 score
Colon	0.9346	0.9250	0.9778	0.9463
ALL1	1.0000	1.0000	1.0000	1.0000
ALL_AML	0.9857	1.0000	0.9667	0.9818
Leukemia	0.9714	1.0000	0.9429	0.9667
Lymphoma	0.9778	1.0000	0.9667	0.9818
Gastric1	0.9717	0.9714	0.9713	0.9709
Gastric2	0.9840	0.9692	1.0000	0.9840
Prostate	0.9405	0.9800	0.9106	0.9437

It can be seen from Table 3 that the proposed method can achieve high classification accuracy, recall, precision and F1 Score on different datasets. All four evaluation indicators on ALL1 can reach the maximum value of 1. Overall, on the eight datasets, BDBA can achieve classification accuracy above 0.93, recall above 0.92, precision above 0.91, and F1 score above 0.94.

**Fig. 3:**
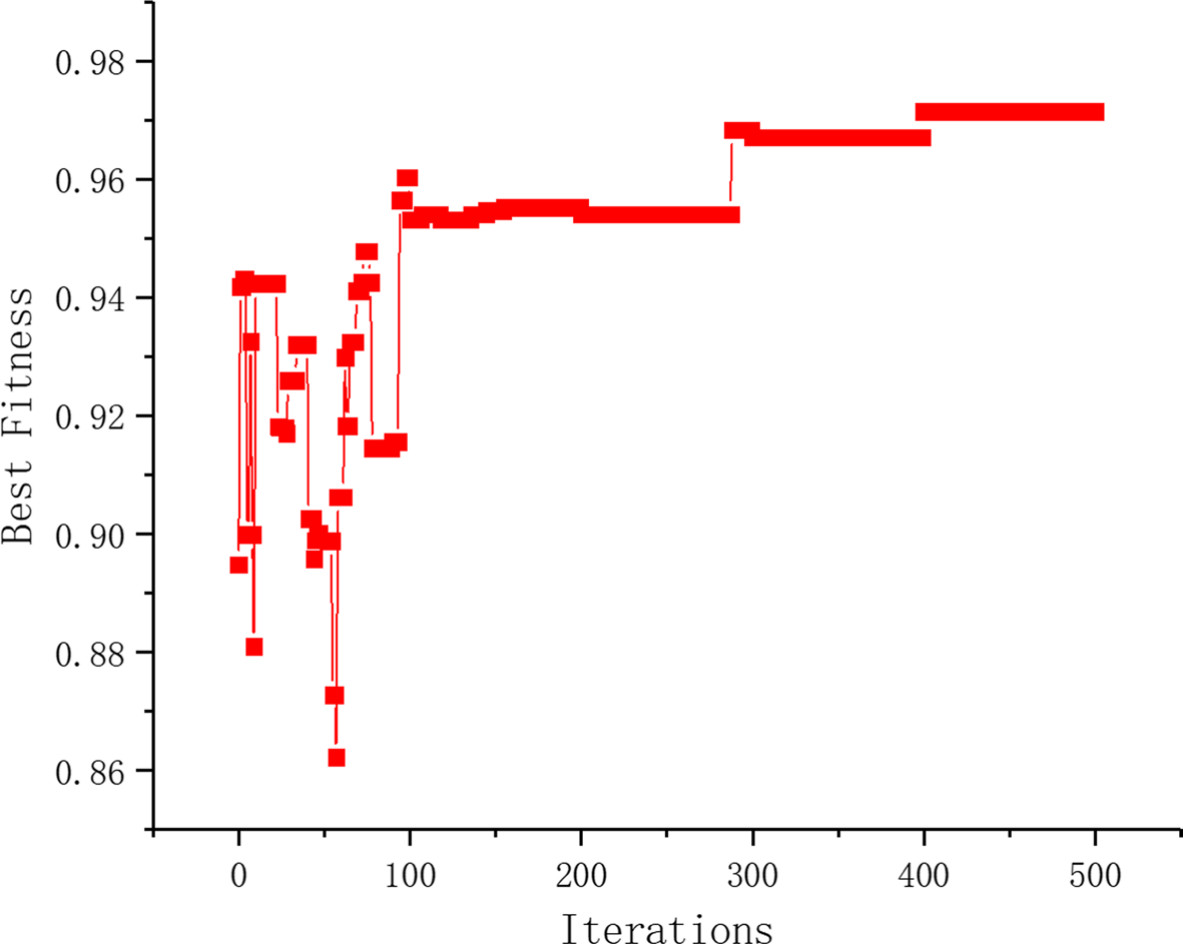
The change of the best individual fitness of each generation in the iterative process of EF-BDBA($\alpha =0.9$) on the Leukemia dataset

To further demonstrate the convergence and usability of EF-BDBA, we take the optimal feature subset obtained on the Leukemia as an example. The fitness changes of the optimal individual in each generation during the iterative process is shown in Fig. 3. It can be seen that the algorithm tends to converge after 500 iterations and obtains the highest fitness.

#### Comparison with traditional feature selection method

**Fig. 4:**
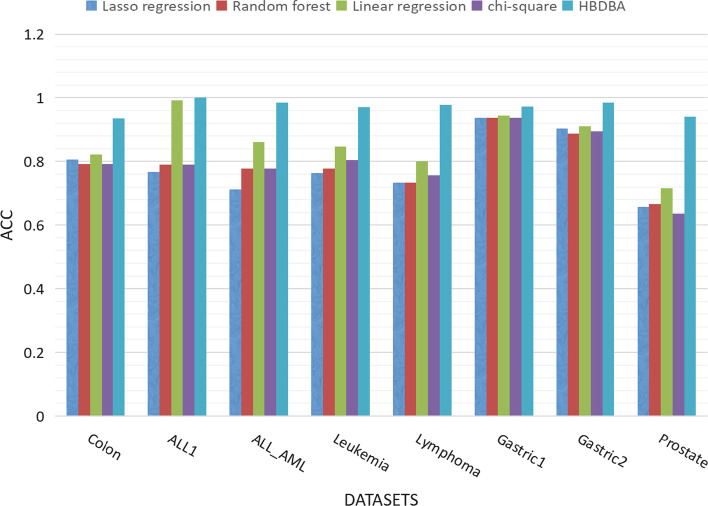
Comparison of EF-BDBA($\alpha =0.9$) and traditional feature selection methods. Lasso regression, random forest, linear regression, and chi-square test were used to select the same number of features as BDBA, respectively, and then the SVM classification model was used to perform five-fold cross-validation on the feature data selected by each model

In the comparison experiment with the traditional feature selection method, we control the traditional method and EF-BDBA to select the feature subset of the same dimension, use the SVM as the classification model and the average classification accuracy of the five-fold cross-validation as the evaluation index. Experimental results indicate the stability and effectiveness of EF-BDBA. The specific experimental results are shown in Fig. 4.

As can be seen from Fig. 4, with the same number of retained features, our proposed method outperforms the other four traditional methods on all datasets. Compared with the average classification accuracy of the other four traditional methods on each dataset, the classification accuracy of our method is improved by $13.21\%$ on Colon, $16.53\%$ on ALL1, $20.40\%$ on ALL_AML, $17.31\%$ on Leukemia, $22.23\%$ on Lymphoma, $3.33\%$ on Gastric1, $8.54\%$ on Gastric2, and $27.14\%$ on Prostate.

#### Comparison with advanced feature selection algorithms

This section compares our method with recently published hybrid feature selection algorithms in terms of the number of selected features and classification accuracy on three widely available public microarray datasets. The detailed comparison results are shown in Table 4.

**Table 4:** Comparison with advanced feature selection algorithms

Datasets	Methods	ACC	Features
Prostate	Wu [17]	0.9160	24.8
	Jinthanasatian [24]	0.8743	5
	Sun [25]	0.8840	4
	Annavarapu [26]	0.8736	8.3
	Khani [27]	0.9216	5
	Wang [28]	0.9040	9
	Xie [23]	0.9124	4
	Our method	0.9405	4
Colon	Wu [17]	0.8833	24.8
	Sun [25]	0.8380	3
	Khani [27]	0.9347	6
	Wang [28]	0.8570	11.1
	Sun [29]	0.8426	5
	Xie [23]	0.9500	4
	Our method	0.9346	3
ALL_AML	Annavarapu [26]	0.9794	3.5
	Khani [27]	0.9822	8
	Our method	0.9857	4
Leukemia	Sun [25]	0.9290	9
	Wang [28]	0.9610	8.3
	Sun [29]	0.8889	4
	Xie [23]	0.9724	5
	Our method	0.9714	3

As can be seen from Table 4, our method has advantages in both the number of retained features and the classification accuracy. Although the classification accuracy of [27] is similar to that of our method on the Colon and ALL_AML datasets, the number of features retained is twice that of our method. The MMBDE method [23] we proposed earlier outperformed obviously the method proposed in this paper only on the Colon dataset. On the Prostate and Lymphoma, the classification accuracy obtained by our method is $2.81\%$ and $2.22\%$ higher than MMBDE, respectively. Therefore, we can still consider the proposed method to be effective and advanced compared to published methods.

#### Statistical analysis of selected biomarkers

In this subsection, we further analyze the features selected by the proposed method, to verify whether the features have the potential as biomarkers from a statistical point of view. The features selected by this method on the ALL1 and Prostate datasets at $\alpha =0.9$ were used as examples for our analysis. Table 5 shows the Prob IDs of the four features selected in each of the two datasets and the corresponding Gene names after the conversion by the GPL platform. Where P-value is the quantitative representation of the results after the T-test for independent samples, * means $P<0.05$, ** means $P<0.01$, and *** means $P<0.001$. The calculation method of T-test is shown in Eq. 1. FC (Fold Change) represents the fold of difference between different samples. The FC is not considered statistically significant, and its value is closer to 1, indicating less variability. The calculation method of FC is shown in Eq. 2.

1 $$\begin{aligned}{} & {} t\left( f_{i}\right) =\frac{\left| {\bar{f}}_{i_{p o s}}-{\bar{f}}_{i_{n e g}}\right| }{\sqrt{S_{i_{p o s}}^{2} / n_{p o s}+S_{i_{n e g}}^{2} / n_{n e g}}} \end{aligned}$$

2 $$\begin{aligned}{} & {} FC=\frac{{\bar{f}}_{i_{p o s}}}{{\bar{f}}_{i_{n e g}}} \end{aligned}$$

Where ${\bar{f}}_{i_{p o s}}$ denotes the mean value of feature $f_{i}$ in the positive sample, ${\bar{f}}_{i_{n e g}}$ denotes the mean value of feature $f_{i}$ in the negative sample. $S_{i_{p o s}}^{2}$ and $S_{i_{n e g}}^{2}$ denote the variance of feature $f_{i}$ in the positive and negative samples, respectively. $n_{p o s}$ and $n_{neg}$ denote the number of samples in the positive and negative samples, respectively.

**Table 5:** Statistical information of selected characteristics on ALL1 and Prostate datasets using the proposed method

Datasets	Prob ID	Gene Name	P-value	FC
ALL1	1110_at	M21624	5.23417E−35 (***)	− 40.58
	34332_at	GNPDA1	1.80973E−19 (***)	0.3423
	40522_at	GLUL	1.55101E−13 (***)	0.5380
	33243_at	TNFAIP8	5.03201E−12 (***)	0.3276
Prostate	38203_at	KCNN1	0.0158853 (*)	1.120
	40282_s_at	CFD	1.52365E−11 (***)	1.646
	32570_at	HPGD	0.000855335 (***)	0.9161
	1060_g_at	NTRK3	0.000113525 (***)	1.467

Where *P*-value is the quantitative representation of the results after the T-test for independent samples

* means *P* < 0.05

** means *P* < 0.01

*** means *P* < 0.001

According to the results in Table 5, for ALL1 data, in the M21624 group, Neg was higher than the average level of Pos, and the difference between the two groups was − 40.58(1.546, − 0.03814), and the difference was statistically significant ($t=17.266$, $P<0.001$). In the GNPDA1 group, Neg was higher than the average level of Pos, the difference between the two groups was 0.3423(0.2026, 0.5918), and the difference was statistically significant ($t=10.726$, $P<0.001$). In the GLUL group, Neg was higher than the average level of Pos, and the difference between the two groups was 0.5380(0.9568, 1.778), and the difference was statistically significant ($t=8.277$, $P<0.001$). In the TNFAIP8 group, Neg was higher than the average level of Pos, the difference between the two groups was 0.3276(0.2577, 0.7877), and the difference was statistically significant ($t=7.630$, $P<0.001$).

For Prostate data, in the KCNN1 group, Neg was lower than the average level of Pos, the difference was 1.120(1.133, 1.012), and the difference was statistically significant ($t = -2.453$, $P = 0.016$). In the CFD group, Neg was lower than the average level of Pos, the difference between the two groups was 1.646(1.944, 1.181), and the difference was statistically significant ($t = -7.614$, $P < 0.001$). In the HPGD group, Neg was lower than the average level of Pos, and the difference between the two groups was 0.9161(− 0.8336, − 0.9099), and the difference was statistically significant ($t = -3.438$, $P = 0.001$). In the NTRK3 group, Neg was lower than the average level of Pos, and the difference between the two groups was 1.467(0.7215, 0.4917), and the difference was statistically significant ($t = -4.019$, $P < 0.001$).

**Fig. 5:**
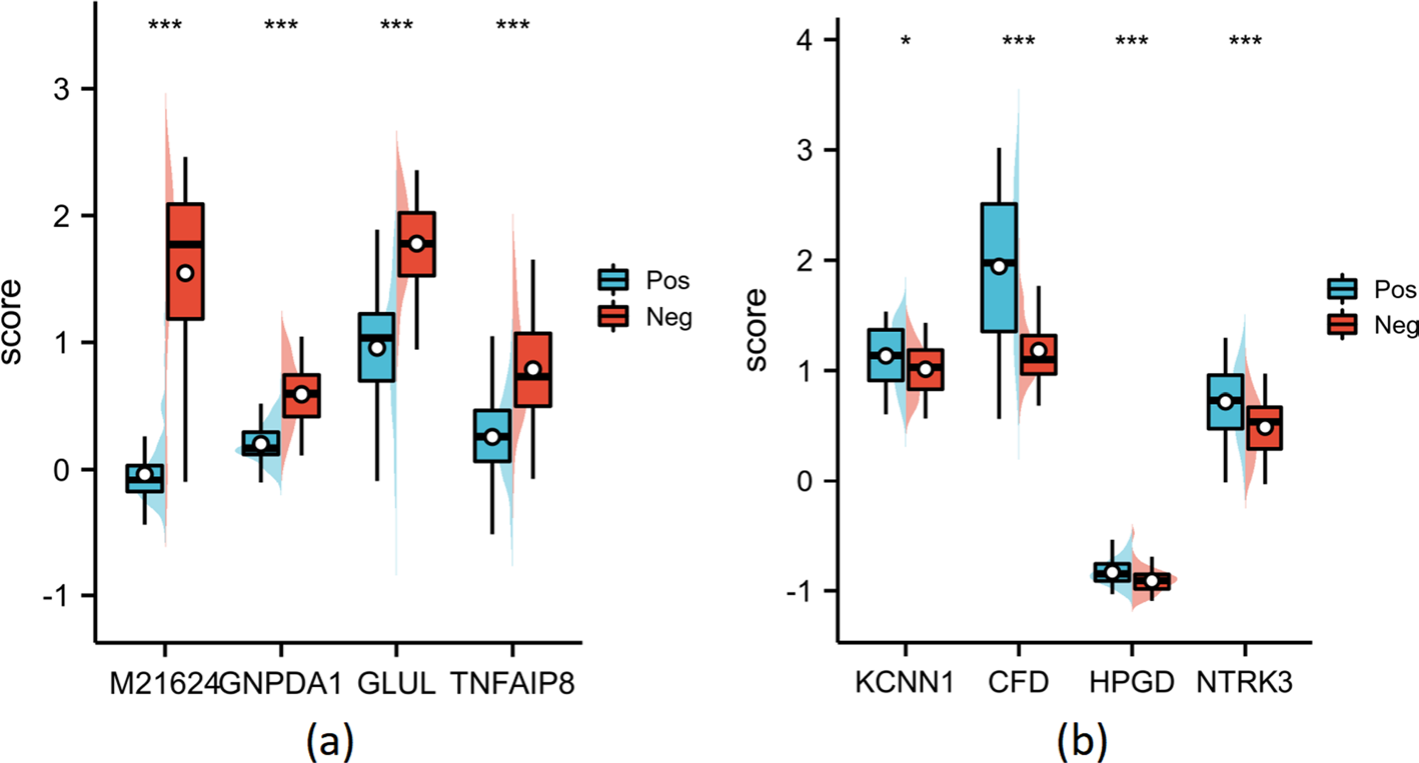
The boxplot of the features selected by the proposed method on positive and negative samples, where **a** represents ALL1 dataset and **b** represents Prostate dataset

**Fig. 6:**
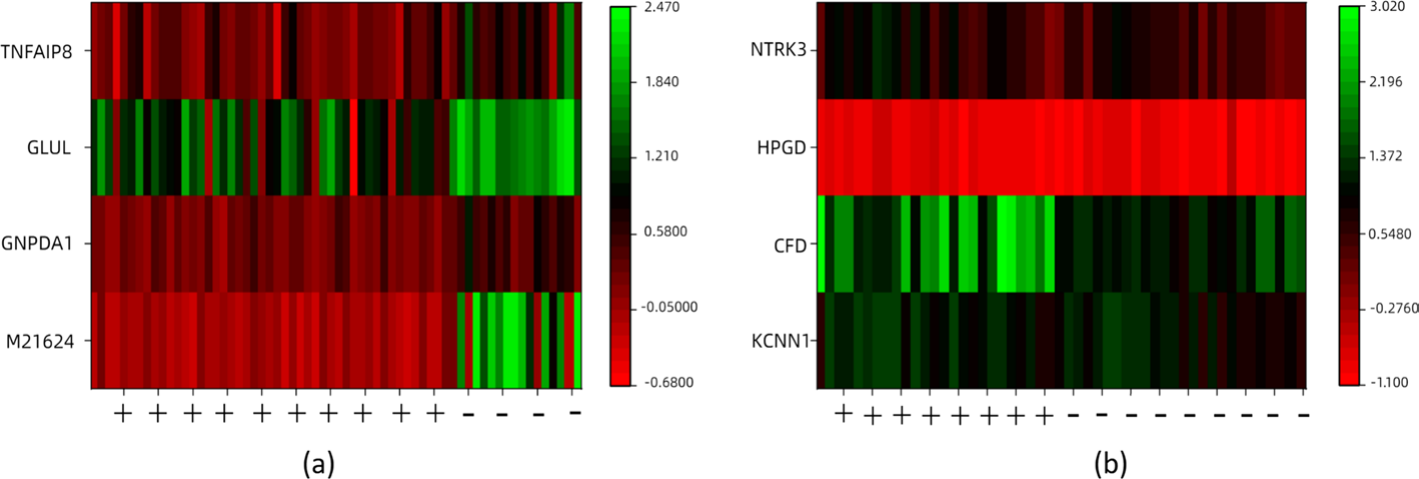
The heat map of the features selected by the proposed method on positive and negative samples, where **a** represents ALL1 dataset and **b** represents Prostate dataset

Figures 5 and 6 show the expression and heat map of the four genes on both positive and negative samples, with Figs. 5a and 6a representing the ALL1 dataset and Figs. 5b and  6b representing the Prostate dataset. As can be seen from the results, the features selected by the proposed method are significantly different on positive and negative samples and can effectively differentiate between positive and negative samples with statistical significance and potential ability to diagnose disease.

**Fig. 7:**
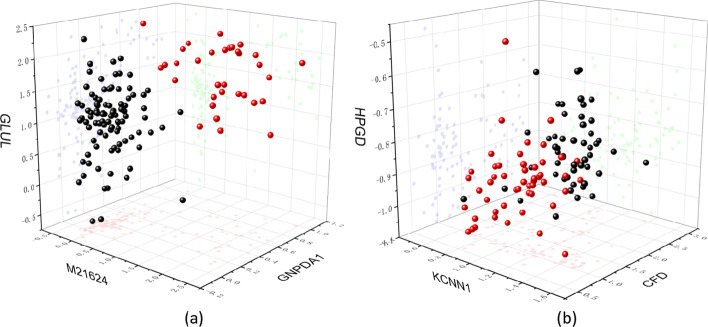
The 3D visualization results of the three features selected by the proposed method, where **a** represents ALL1 dataset and **b** represents Prostate dataset. The red and black balls represent positive and negative samples, respectively

In order to visualize the features selected by the proposed method more clearly, we selected three features on each dataset for 3D visualization, and the results are shown in Fig. 7. Figure 7a represents the ALL1 dataset and Fig. 7b represents the Prostate dataset. The three axes represent different features, and different colors represent different samples. It can be seen that in the three-dimensional space, the features selected by the proposed method have significant differences in the expression of different samples, which indicates that these features can effectively distinguish positive and negative samples and have diagnostic significance.

**Fig. 8:**
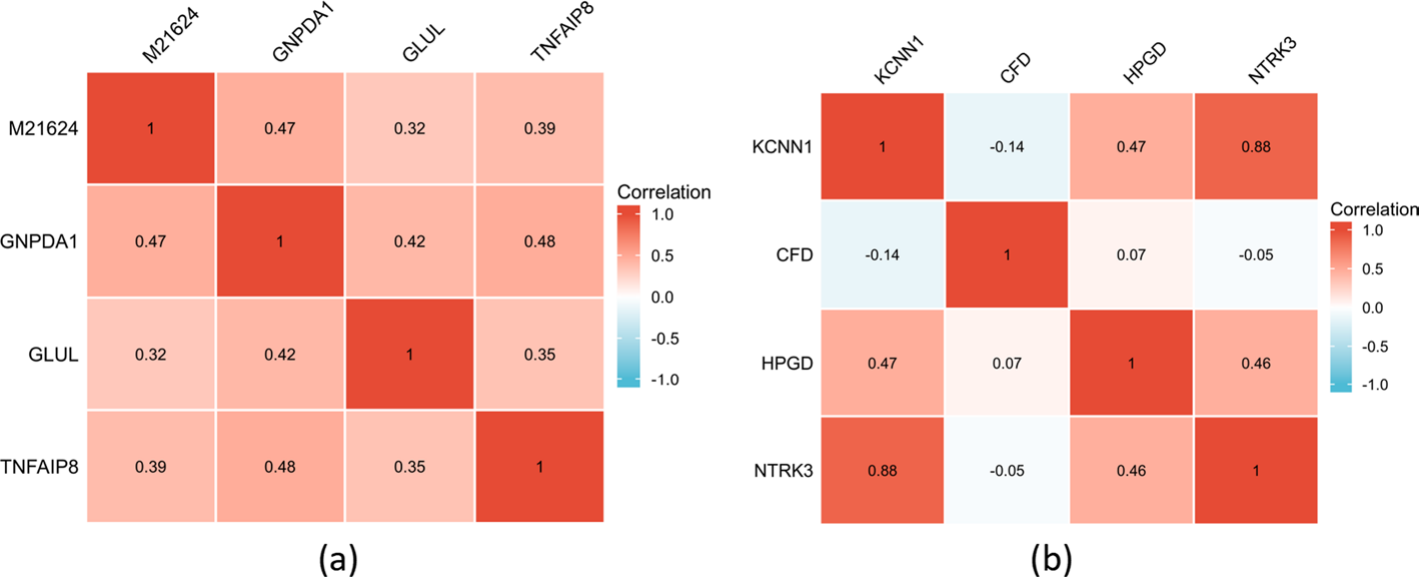
Correlation analysis was performed using Spearman’s correlation coefficient for the four selected features, where **a** represents ALL1 dataset and **b** represents Prostate dataset

Figure 8 shows the correlation analysis of the features selected by the proposed method. We use the Spearman correlation coefficient as an example for the analysis, and it can be seen from the results that none of the features selected by the proposed method are significantly correlated in the ALL1 dataset, while on the prostate dataset, only one set of features is correlated and the rest are not significantly correlated, which proves that the features selected by the proposed method have a low redundancy.

## Discussion

From Fig. 2, it can be seen that in most of the datasets, comparing with the single fisher score method, the quality of the feature pool obtained by the ensemble filter method is better. The ensemble filter method proposed considers inter-class distance and intra-class distance, while measuring the correlation between features and class labels, redundancy and complementarity between features. Dedicated to filtering irrelevant features, it provides an excellent feature pool for downstream wrapped methods.

From Table 3, it can be seen that on the eight public datasets, our method obtained high scores on four conventional metrics. As can be seen from Fig. 4, compared with the traditional feature selection methods, the proposed method has an advantage among all the eight public datasets. Meanwhile, as shown in Table 4, our method achieves higher classification accuracy with fewer features than the advanced feature selection methods in recent years. It implies that the prediction of the corresponding diseases can be well achieved with fewer target biomarkers.

In Table 5, we statistically validate the potential of the selected features as biomarkers. In Figs. 5, 6, 7 and 8, we further verified the effectiveness of these features. It can be seen from the figures that the feature subset we identified can effectively distinguish between positive and negative samples. On the whole, the proposed method can effectively identify excellent feature subset, namely valuable biomarkers, and the experimental results demonstrate the efficiency of the proposed method.

While our method obtains excellent results, it also has certain limitations. From Fig. 2, it can be seen that the advantage of the ensemble filter method is not obvious in some datasets. It may be due to the fact that we utilize prior knowledge in the improved fast correlation-based filter (FCBF) algorithm. This is subjective to a certain extent, which affects the quality of the feature pool. In addition, our proposed method is a single-objective feature selection method, which combines the number of retained features with the classification accuracy as an fitness function. Compared with the multi-objective feature selection algorithm, the design of the single-objective algorithm has a slight impact on the stability of the results.

In the future work, we will continue to improve the measurement mechanism of the ensemble filter method. It avoids subjective factors on the quality of the feature pool and makes the method more robust and stable. Meanwhile, we will also study the multi-objective feature selection algorithm based on EF-BDBA, in which the number of selected features and the classification accuracy are used as two independent objectives to search for the optimal solution, thereby obtaining more stable and meaningful biomarkers.

## Conclusion

In this paper we propose a two-stage mixed feature selection algorithm for gene microarray data. In the first stage, an ensemble filter feature selection method is designed to perform preliminary dimensionality reduction on the microarray data, so as to provide an excellent candidate feature pool for the downstream wrapped method. In the second stage, we designed a hybrid warpped method. Integrating the improved binary differential evolution algorithm and the binary African vulture optimization algorithm effectively balances the exploration and exploitation capabilities of the algorithm. The optimal feature subset with high classification accuracy and small dimension can be selected from the candidate feature pool. The experimental results demonstrate the effectiveness and advanced of our proposed algorithm model.

## Method

### Datasets

Eight publicly available datasets used in the experiment are from the GEO (Gene Expression Omnibus) database https://www.ncbi.nlm.nih.gov/geo/ and http://csse.szu.edu.cn/staff/zhuzx/Datasets.html, namely ALL1, ALL_AML, Colon, Leukemia, Lymphoma, Gastric1, Gastric2 and Prostate. These datasets are typical microarray data. The detailed dataset information is shown in Table 6. The number of features in each dataset is much larger than the number of samples. For example, the number of features exceeds 120 times the number of samples in the Prostate dataset. Moreover, except for the Gastric1 and Gastric2 datasets, the number of positive and negative samples in the rest of the datasets is not equal.

**Table 6:** Microarray data

Datasets	Samples	Features	Category Distribution
Colon	62	2000	Tumor: 40, Normal: 22
ALL1	128	12,625	B-cell: 95, T-cell: 33
ALL_AML	72	7129	AML: 25, ALL: 47
Leukemia	72	7129	AML: 25, ALL: 47
Lymphoma	45	6937	ACL: 23, GCL: 22
Gastric1	144	22,283	N: 72, T: 72
Gastric2	124	22,283	N: 62, T: 62
Prostate	102	12,625	Tumor: 52, Normal: 50

### The overall framework of the proposed method

**Fig. 9:**
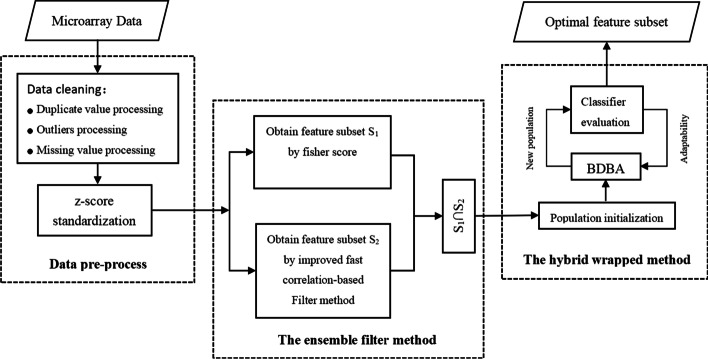
The overall framework of the feature selection algorithm is described in this figure. The process includes data preprocessing, an ensemble filter method to perform initial dimensionality reduction of the data, and a hybrid wrapped method called BDBA to obtain the final optimal feature subset

In this section, we mainly introduce the two-stage feature selection algorithm proposed in this paper. The overall framework of the algorithm is shown in Fig. 9. The original data is preprocessed before feature selection. The specific operations include deduplication, using Tukey’s test to detect outliers, and using the KNN model to fill in outliers and missing values. Then the z-score normalization is performed on the cleaned data to remove dimensional effects between different feature data. Next, a two-stage feature selection operation is performed on the preprocessed data. In the first stage, using the ensemble filter method integrating the improved fast correlation-based filter algorithm and fisher score to perform initial dimensionality reduction on the data, and the intersection of the two feature subsets selected by the improved fast correlation-based filter algorithm and fisher score is used as the candidate feature pool input to the downstream BDBA. In the second stage, the population is randomly initialized, BDBA is used to evolve the population, and then the classifier is used to evaluate the quality of individuals in the new generation population until the maximum number of iterations is reached, and the optimal feature subset is output.

### The Pre-processing method

The original data usually contains noise, which will inevitably affect the performance of the algorithm and cause errors in the experimental results. Therefore, we need to clean and standardize the data. In the data cleaning stage, the repeated features in the data are averaged first, then Tukey’s test is used to detect outliers in the data, and the KNN model is used to fill in outliers and missing values. Then, z-score normalization is performed on the cleaned data to remove dimensional effects between different feature data.

### The proposed ensemble filter method

The classic FCBF algorithm uses symmetric uncertainty (SU) to measure the correlation between features and class labels, and the redundancy between features. First, an appropriate threshold is set to eliminate features with weak correlation to obtain a subset of candidate features. Then, in each round of iteration, the feature with the largest correlation in the candidate feature subset is taken out of the candidate set as a salient feature and added to the optimal feature subset. Next, the approximate Markov blanket principle is applied to eliminate other features in the candidate set with high redundancy with the salient features, until the candidate set is empty, then the final optimal feature subset is obtained [30]. Compared with the traditional Correlation-Based Filter algorithm [31], FCBF does not need to calculate the correlation between each pair of features, so it greatly improves the efficiency of feature selection, but the algorithm ignores the complementarity between features, and SU is an entropy-based correlation measurement method, which is more suitable for processing discrete data and cannot be directly applied to continuous gene microarray data. Therefore, we propose the improved FCBF algorithm, design a complementarity measure method based on Manhattan distance, and use the Pearson correlation coefficient [32] instead of SU for continuous microarray data to quantitatively estimate the correlation between features and class labels, and the redundancy between features. Algorithm 1 describes the process of the improved FCBF.
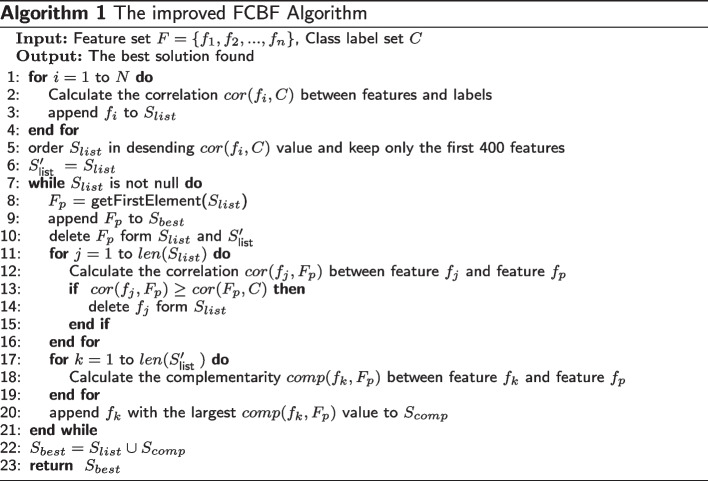


The complementarity between features is also called information collaboration or information interaction, which indicates that the classification information that two features can provide by working together may be greater than the sum of the information contained in each of the two features [33]. It can be seen from Algorithm 1 that after removing redundant features in each iteration, The imporoved FCBF will select the feature with the greatest complementarity with the salient feature from the feature set to be eliminated and add it to the complementary feature set. Inspired by the idea of instance similarity used by ReliefF algorithm, Sun proposed that more attention should be paid to the distance between an instance and its heterogeneous nearest neighbor in different feature spaces in order to analyze the classification and prediction ability after feature combination [34]. Inspired by Sun’s idea, in order to calculate the complementarity between two features, we first randomly select a sample $S_i$ in the sample space, and calculate the manhattan distance between $S_i$ and its heterogeneous nearest neighbor in two different feature dimensions, and the heterogeneous nearest neighbor is the heterogeneous sample adjacent to the $S_i$ index in the sample space. In order to ensure the stability of the obtained results, we randomly select n different samples for calculation, and then average the results. The calculation method of the complementarity between features is shown in Eq. 3.

3 $$\begin{aligned}{} & {} C=\frac{\sum d i s}{n} \end{aligned}$$

4 $$\begin{aligned}{} & {} {\text {dis}}=\left| v_{i p}-v_{j p}\right| +\left| v_{i q}-v_{j q}\right| \end{aligned}$$

In Eq. 3, C represents the degree of complementarity between the feature $f_p$ and the feature $f_q$. *dis* represents the manhattan distance between the sample $S_i$ and its heterogeneous nearest neighbor $S_j$ on the feature dimension $f_i$ and the feature dimension $f_j$, which is calculated using Eq. 4. In Eq. 4, $v_{ip}$ and $v_{iq}$ represent the values of the sample $S_i$ on the feature dimensions $f_p$ and $f_q$, respectively. $v_{jp}$ and $v_{jq}$ represent the values of the sample $S_j$ on the feature dimensions $f_p$ and $f_q$, respectively.

The pearson correlation coefficient is used to quantify and estimate the correlation between features and category tags and the redundancy between features. The calculation method of the pearson correlation coefficient is shown in Eq. 5.

5 $$\begin{aligned} r=\frac{\sum \left( x-m_{x}\right) \left( y-m_{y}\right) }{\sqrt{\sum \left( x-m_{x}\right) ^{2} \sum \left( y-m_{y}\right) ^{2}}} \end{aligned}$$

where *r* represents the correlation coefficient, and $m_x$ and $m_y$ represent the mean of the vectors *x* and *y*, respectively. When calculating the correlation between features, *x* and *y* correspond to two different feature vectors, respectively. We refer to the specific operation of T-test when filtering features that are not related to class labels [35]. When calculating the correlation between features and class labels, *x* and *y* correspond to the vector $f_{pos}$ on positive samples and the vector $f_{neg}$ on negative samples, respectively. Due to the imbalance of samples in the dataset, the dimensions of $f_{pos}$ and $f_{neg}$ may be different, which cannot be calculated directly using Eq. 3. Therefore, we first approximately fill the unbalanced data through the SMOTE oversampling technique. The principle of SMOTE is to select a random sample $S_j$ in its nearest neighbors for each minority class sample $S_i$ [36], and then synthesize a new minority class sample $S_k$ through Eq. 6.

6 $$\begin{aligned} s_{k}=s_{i}+{\text {rand}}(0,1)^{*}\left| s_{i}-s_{j}\right| \end{aligned}$$

Since the greater the correlation between the positive and negative sample vectors of the feature, the smaller the correlation between the feature and the class label, and the range of the Pearson correlation coefficient is known to be between 0 and 1, so we use Eq. 7 to quantify the estimate correlations between features and class labels.

7 $$\begin{aligned} \textrm{r}_{c f}=0.8 *\left( 1-r_{p n}\right) \end{aligned}$$

where $r_{cf}$ represents the correlation between features and class labels, and $r_{pn}$ represents the correlation between the positive and negative sample vectors corresponding to the features obtained by Eq. 3. The larger the $r_{cf}$, the smaller the probability of being deleted as redundant features. In order to prevent the deletion of meaningful features by mistake, we set a weight coefficient of 0.8 for it.

In order to avoid the unstable results of a single correlation-based filter method, fisher score is also used to select the top 100 features in the dataset, and finally the intersection of the two feature subsets obtained by the improved FCBF and fisher score is provided to the downstream wrapped method. fisher score is an effective filter feature selection method. Its core concept is that the features with strong discriminative performance are shown as small intra-class distances as possible, and inter-class distances as large as possible [37]. The calculation method of fisher score is shown in Eq. 8.

8 $$\begin{aligned}{} & {} \textrm{J}_{\text{ fisher } }(x)=\frac{S_{B}^{x}}{S_{\omega }^{x}} \end{aligned}$$

9 $$\begin{aligned}{} & {} S_{B}^{x}=\sum _{i=1}^{C} \frac{n_{i}}{n}\left( m_{i}^{x}-m^{x}\right) ^{2} \end{aligned}$$

10 $$\begin{aligned}{} & {} S_{\omega }^{x}=\frac{1}{n} \sum _{i=1}^{C} \sum _{y \in \omega _{i}}\left( y^{x}-m_{i}^{x}\right) ^{2} \end{aligned}$$

In Eq. 8, $J_{fisher}(x)$ represents the fisher score of feature *x*, $S_B^x$ represents the inter-class variance of feature *x* on the dataset, the calculation method is shown in Eq. 9, $S_{\omega }^{x}$ represents the intra-class variance of feature *x* on the dataset, and the calculation method is as Eq. 8 shown. In Eq. 9, *n* represents the number of samples, $n_i$ represents the number of samples of the i-th type, $m_i^x$ represents the mean of the i-th type of samples on the feature dimension *x*, and $m^x$ represents the mean of all samples on the feature dimension *x*. In Eq. 10, $y^x$ represents the value of the sample *y* on the feature dimension *x*.

### The proposed hybrid wrapped method

Differential evolution was first proposed by Storn and Price in 1997 [38]. It is a population-based meta-optimization algorithm with simple structure, fast convergence, and strong robustness. The evolution operator of the algorithm also has three main steps: mutation, crossover and selection. In order to achieve the goal of controlling the number of selected features and speeding up the convergence speed of the algorithm, this paper proposes an improved binary differential evolution algorithm (IBDE), redesigns the evolution operator of the binary differential evolution algorithm, and proposes a segmental adaptive fitness calculation method. However, in the process of evolution, even if we try to balance the exploration and exploitation capabilities of the algorithm in the design of the crossover factor and the scaling factor, in order to control the number of retained features, the diversity of the early population is inevitably weakened, so we combine the AVOA with excellent global optimization ability.

AVOA simulates the foraging and navigation behavior of African vultures. The vultures determine different foraging strategies based on the hunger level they feel. The algorithm can be divided into four independent stages: in the first stage, determine the best vulture; in the second stage, the hunger rate of the vultures is calculated; the third stage is the exploration stage, which includes updating the individual position in two ways: moving randomly or approaching the best vulture at a random distance; the fourth stage is the exploitation stage, including the transitional stage of updating individual positions in a siege-fight or rotating flight, and the later exploitation stage of approaching outstanding individuals in a more drastic way. Details of the above movement strategies are available in the paper [39].

It is worth noting that the individual position update in AVOA does not completely and independently enter the third and fourth stages in sequence, but adds a certain exploitation mechanism in the early exploration process through the starvation rate, which avoids AVOA from falling into local optimality, and accelerates the convergence of the algorithm while ensuring that the AVOA does not diverge too much. In addition, the setting of the transition stage can make the population evolution proceed smoothly and improve the optimization performance of the algorithm. However, AVOA is more suitable for dealing with engineering problems of searching for optimal solutions in continuous space. Feature selection for microarray data is a solution optimization problem in discrete space, in which individuals need to be represented by binary vector codes (1 means the feature is selected, 0 means the feature is not selected). Therefore, we propose a binary african vultures optimization algorithm (BAOVA) to binary quantify AVOA. In addition, the optimization process of AVOA can be briefly understood as the process of increasing the degree of approaching the best vulture. However, AVOA’s selection of the best vulture is limited to the two vultures with the first and second fitness, which will have a negative impact on the algorithm’s early exploration ability. For this reason, in BAOVA, we use the opposition-based Learning (OL) method to obtain new individuals to select the best vultures for AVOA.

**Fig. 10:**
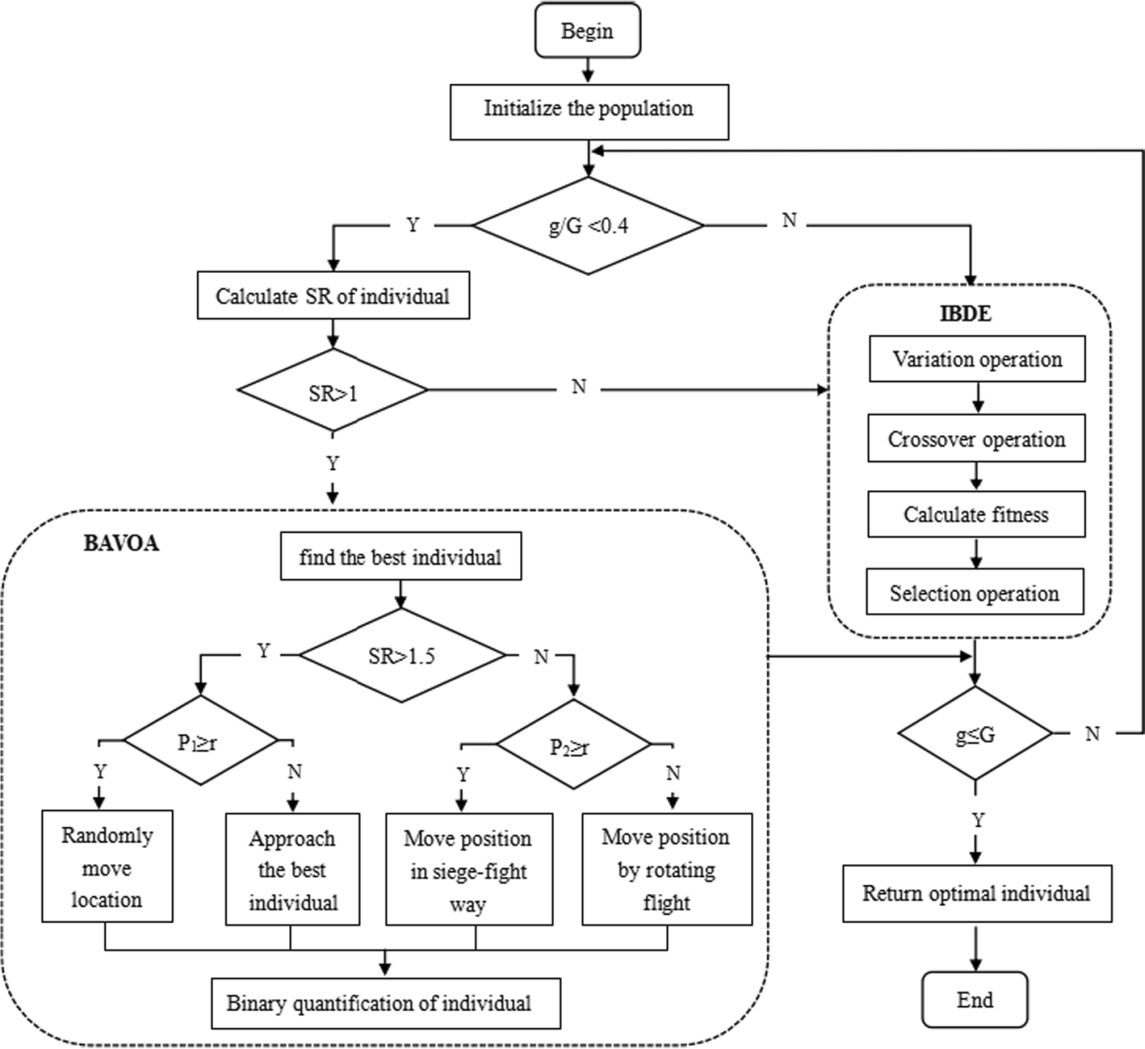
Flowchart of BDBA. Randomly initialize the population. Then, in the first $40\%$ of the iterative process, use the hunger rate strategy in AVOA to integrate BAVOA into IBDE, when the individual’s hunger rate $SR<1$, use BAVOA to update the individual, otherwise use IBDE to update the individual. During the last $60\%$ of the iterative process, IBDE is used to evolve the population to find the optimal individual

How to effectively combine IBDE with BAVOA and establish a stable, robust and efficient overall algorithm model is an important issue. BAVOA is used to enhance the exploration ability of the algorithm. In BDBA, if the proportion of BAVOA is too small, it will not be able to play its role effectively, and if the proportion of BAVOA is too large, it will affect the local development ability of BDBA, which will easily lead to slow algorithm convergence, and it is difficult to find excellent feature subset. We refer to the strategy of combining bat algorithm and differential evolution in the paper [40]. And we try to fuse BAVOA for IBDE when the condition $g / G<0.4$ is satisfied, where *G* represents the maximum number of iterations and *g* represents the current number of iterations. The algorithm flow of BDBA is shown in Fig. 10. Referring to the good design of AVOA adding the exploitation mechanism in the exploration phase, we also use the same method to integrate IBDE and BAVOA, and retain the individual location update strategy in the exploration phase and transition phase of AVOA. BAVOA was conditionally added to the evolution of IBDE using the starvation rate *SR* designed in AVOA. The BDBA’s flowchart is shown in Fig. 10.

#### Parameter setting of BDBA method

Since improved binary AVOA uses the basic framework of AVOA, the parameters $P_1$ and $P_2$ are consistent with AVOA. If the value of *G* is too large, it will waste computing resources. We set it to 500 under the condition that the algorithm converges. The larger the value of *NP*, the greater the population diversity, but too large *NP* will cause the algorithm to converge slowly, which is not conducive to the local optimization of the algorithm. According to the size of the input feature pool, we set it to 30. The experimental parameters of BDBA are shown in Table 7.

**Table 7:** BDBA algorithm parameters

Parameters	Values	Description
G	500	The maximum number of iterations
NP	30	Population size
$P_1$	0.6	The first probability parameter of the selection mechanism in BAVOA
$P_2$	0.4	The second probability parameter of the selection mechanism in BAVOA
$\alpha$	0.9	Adaptive cross factor parameter

#### Improved binary differential evolution

In the mutation stage of IBDE, we propose a new mutation operator, which can effectively control the number of retained features and improve the convergence speed of the algorithm. The calculation method of the difference vector diff $_{i}^{g}$ of the individual $x_i^g$ is shown in Eq. 11.

11 $$\begin{aligned}{} & {} {\text {diff}}_{i, j}^{g} = \left\{ \begin{array}{ll} 0, &{} \text{ if } x_{r 1, j}^{g} = x_{r 2, j}^{g} \\ F x_{r 1, j}^{g}, &{} \text{ otherwise } \end{array}\right. \end{aligned}$$

12 $$\begin{aligned}{} & {} \textrm{F}=f_{min} + \left( \left( G-g+1\right) /g\right) * \left( f_{max} - f_{min}\right) \end{aligned}$$

where $x_{r1,j}$ and $x_{r2,j}$ represent the values on the j-th dimension of the two individual vectors randomly selected in the g-th iteration. *F* is the inertial adaptive scaling factor, which is calculated by Eq. 12. In Eq. 12, $f_{min}$ and $f_{max}$ are the minimum and maximum values that can be achieved by *F*, respectively. Since the value in the individual vector is binary, diff$\in [0, F]$. In order to perform the downstream mutation operation, it is necessary to control F$\in [0, 1]$. At the same time, in order to increase the randomness and not to choose or abandon a certain feature absolutely, we set the values of $f_{min}$ and $f_{max}$ to 0.1 and 0.9, respectively. The *F* gradually decreases as the number of iterations increases. The larger *F*, the better for the global search, and the smaller *F* is for the local search. In this way, the ability of algorithm exploration and exploitaion can be balanced to a certain extent. The calculation method of the mutation vector $u_i^g$ of the individual $x_i^g$ is shown in Eq. 13.

13 $$\begin{aligned}{} & {} u_{i, j}^{g} = \left\{ \begin{array}{ll} 1, &{} \text{ if } \text{ pr } \ge {\text {rand}}(0,1) \text{ and } x_{r 3, j}^{g} = 1 \\ 0, &{} \text{ otherwise } \end{array}\right. \end{aligned}$$

14 $$\begin{aligned}{} & {} pr = \frac{e^{{\text {diff}}_{i, j}^{g}}-e^{-diff_{i, j}^{g}}}{e^{{\text {diff}}_{i, j}^{g}}+e^{-diff_{i, j}^{g}}} \end{aligned}$$

In Eq. 13, $x_{r3.j}^g$ represents the value of the j-th dimension in the random individual vector in the g-th iteration. *pr* is used to ensure that the final binary vector is close to 0 or 1. The calculation method of *pr* is shown in Eq. 14.

In the crossover stage, we propose an adaptive crossover factor, which aims to gradually improve the convergence ability of the algorithm during the evolution. The calculation method of *CR* is shown in Eq. 15.

15 $$\begin{aligned} C R=\alpha \frac{2 e^{-(g / G)}}{e^{(g / G)}+e^{-(g / G)}} \end{aligned}$$

where $\alpha$ is the adaptive cross factor parameter and *G* is the maximum number of iterations. We can obtain the trial vector $v_i^g$ of individual $x_i^g$ by *CR*, and the calculation method is shown in Eq. 16.

16 $$\begin{aligned} v_{i, j}^{g}=\left\{ \begin{array}{ll} u_{i, j}^{g}, &{} \text{ if } {\text {rand}}(0,1) \le \textrm{CR} \text{ or } j=\textrm{jrand} \\ x_{i, j}^{g}, &{} \text{ otherwise } \end{array}\right. \end{aligned}$$

It can be seen from Eq. 16 that the larger the CR, the higher the probability of an individual accepting the variation. Conversely, the probability of rejecting the mutation and maintaining the individual as they are is higher. Finally, the selection operator decides whether to keep the target vector $x_i^g$ or the trial vector $v_i^g$ to enter the next iteration. The calculation of the selection operator is shown in Eq. 17.

17 $$\begin{aligned} x_{i}^{g+1}=\left\{ \begin{array}{ll} v_{i}^{g}, & {\text{if}}\; f\left( v_{i}^{g}\right) \text{ betther } \text{ than } f\left( x_{i}^{g}\right) \\ x_{i}^{g}, & \text{ otherwise } \end{array}\right. \end{aligned}$$

where $f\left( v_{i}^{g}\right)$ and $f\left( x_{i}^{g}\right)$ represent the fitness of the target vector $x_i^g$ and the trial vector $v_i^g$, respectively. In order to control the number of retained features, we not only use the five-fold cross-validation classification accuracy of SVM as the evaluation standard, and the number of retained features is added to the fitness function as an evaluation condition. The fitness function is calculated as shown in Eq. 18.

18 $$\begin{aligned}{} & {} f(x)=\left\{ \begin{array}{ll} {\text {acc}}-\beta (\textrm{num} / L), &{\text{if}}\; \beta <0.9 \\ a c c, &{\text{otherwise}} \end{array}\right. \end{aligned}$$

19 $$\begin{aligned}{} & {} \beta =\left( \left( \lfloor g / 100\rfloor ^{*} 100\right) / G\right) +0.1 \end{aligned}$$

In Eq. 18, *acc* is the five-fold cross-classification accuracy rate obtained from the microarray data corresponding to the individual vector *x*, *num* is the number of retained features, *L* is the individual dimension, and $\beta$ is the weight coefficient. After every 100 iterations, the value of $\beta$ also increases in sections. When the value is greater than 0.9, it indicates that the iteration has entered the final stage, so we should focus on improving the classification accuracy, and no longer need to control the number of retained features, so let $\beta = 0$.

#### Improved binary African vultures optimization algorithm

OL can effectively make the offspring obtain better solutions [41]. We propose a random OL method to process the individual $x_{first}$ with the largest fitness, and the obtained new individual $x_{new}$ is used to replace the individual with the second fitness in AVOA. The specific operation of the random OL method is to invert the random bits of $x_{first}$, and the number of inverted bits is half of the dimension of $x_{first}$. In this way, not only is the newly obtained individual better than $x_{first}$ in a certain probability, but also the obtained new individual is random, which is beneficial to improving the diversity of the population. Then, the best individual $x_{best}$ is selected between the $x_{first}$ and $x_{new}$ using Eq. 20.

20 $$\begin{aligned} x_{b e s t}=\left\{ \begin{array}{ll} x_{\text{ first } }, &{\text{if}}; {\text {rand}}(0,1)<=f\left( x_{\text{ first } }\right) \\ x_{n e w}, & \text{ otherwise } \end{array}\right. \end{aligned}$$

where $f\left( x_{\text{ first } }\right)$ represents the fitness of $x_{first}$, and the calculation method is shown in Eq. 18.

The method of AVOA algorithm to update the random position of vultures in the exploration stage is not suitable for the calculation of binary vectors, so we have improved it, as shown in Eq. 21.

21 $$\begin{aligned}{} & {} x_{i}^{g+1}=x_{i}^{g}+D^{*} S R \end{aligned}$$

22 $$\begin{aligned}{} & {} D=(2 * {\text {rand}}(0,1)) * x_{r 1}^{g}-x_{\textrm{r} 2}^{g} \end{aligned}$$

In Eq. 22, $x_{r1}^g$ and $x_{r2}^g$ represent two individual vectors randomly selected in the g-th iteration.

As mentioned earlier, the vulture positions updated by AVOA get continuous solutions, so we need to discretize these continuous solutions. The transformation is achieved by using the sigmoid function to compress the continuous decompression in each dimension. In order to better conform to the movement mechanism of the individual during the transformation process, we perform a translation operation on the sigmoid function. The calculation method is shown in Eq. 23.

23 $$\begin{aligned} s=\frac{1}{1+e^{-x_{i, j}^{g}-2}} \end{aligned}$$

where $x_{i,j}^g$ represents the value of the j-th dimension of the i-th individual in the g-th iteration. The solution obtained by the sigmoid function is still continuous, as shown in Eq. 24, in order to better binary quantize the continuous solution, we generally apply a random threshold on the basis of the sigmoid function to obtain the binary solution.

24 $$\begin{aligned} x_{i, j}^{g+1}=\left\{ \begin{array}{ll} 1, &{\text{if}}\; {\text {rand}}(0,1)>s \\ 0, &{\text{ otherwise}} \end{array}\right. \end{aligned}$$

## Data Availability

The public data set used in our experiment is from the GEO (Gene Expression Omnibus) database, which can be obtained through the following website:https://www.ncbi.nlm.nih.gov/geo. https://github.com/xwdshiwo/BioFSDatasets.

## Abbreviations

FCBF: Fast correlation-based filter
AVOA: The African vulture optimization algorithm
BAVOA: The binary African vulture optimization algorithm
IBDE: The improved binary differential evolution algorithm
SVM: The support vector machine
SU: Symmetric uncertainty
OL: The opposition-based Learning
